# Optimization of a high-performance lead-free cesium-based inorganic perovskite solar cell through numerical approach

**DOI:** 10.1016/j.heliyon.2022.e11719

**Published:** 2022-11-17

**Authors:** Tasmin Kamal Tulka, Nowshin Alam, Md Akhtaruzzaman, K. Sobayel, M. Mofazzal Hossain

**Affiliations:** a Department of Mathematics and Natural Sciences, BRAC University, Dhaka, Bangladesh; b Department of Electrical and Electronic Engineering, American International University-Bangladesh, Dhaka, Bangladesh; c Solar Energy Research Institute, The National University of Malaysia, 43600 Bangi, Selangor, Malaysia; d Department of Electrical and Electronic Engineering, University of Liberal Arts Bangladesh, Dhaka 1207, Bangladesh

**Keywords:** CsGeI_3_ perovskite, ETL, HTL, ZnOS, CZTSe, Clean energy, SCAPS-1D

## Abstract

In this work, an ultra-thin (0.815 μm) lead-free all-inorganic novel PV cell structure consisting of solid-state layers with the configuration SnO_2_/ZnOS/CsGeI_3_/CZTSe/Au has been optimized using SCAPS-1D simulator. ZnOS electron transport layer (ETL) has been deployed and various hole transport layer (HTL) material candidates have been considered to find the most suitable one in order to get the maximum possible power conversion efficiency *(PCE)*. The simulation begins with the optimization of the thickness of the ZnOS buffer layer, followed by an analysis of HTL and ETL doping concentrations, thickness and bandgap optimization of absorber layer. The maximum permissible defect density at the ZnOS/CsGeI_3_ interface and the bulk defect density of the absorber layer (CsGeI_3_) are also investigated. It is also found that when the temperature rises, short circuit current density (*J*_*sc*_) rises by 1.43 mA/K and open-circuit voltage (*V*_*oc*_*)* degrades by 2 mV/K. The optimized structure results in a *PCE* of 26.893% with *J*_*sc*_, *V*_*oc*_, and fill factor (*FF*) of 28.172 mA cm^−2^, 1.0834 V, and 88.107% respectively. The cell performance parameters outperform those found in the recent literature. The simulated results of the proposed configuration are expected to be a helpful reference for the future implementation of a cost-effective and efficient all-inorganic perovskite PV cell.

## 1 Introduction

For the sake of transitioning to clean energy, solar cell researchers have endeavored for many years to develop economical, sustainable and environment-friendly materials that offer good optoelectronic properties. As the widely studied inorganic semiconductor solar cells offer relatively higher cost and are limited in efficiency, the use of organic-inorganic lead halide perovskite materials as absorber material have become more prevalent in photovoltaic cells in recent years [1, 2]. Perovskite materials have an ABX_3_ structure where A is a monovalent organic cation (methylammonium, formamidinium, Cs, Rb), B is an inorganic metal cation (Pb, Sn) and the X-is a halide anion (Cl, Br, I) [3]. Since its first application in dye-sensitized solar cell by Kojima et al [4], perovskite materials have been researched by many scientists due to their electrical and optical characteristics suitable for solar cell design, such as tunable bandgap, relatively high carrier mobility and high absorption coefficients in the visible solar spectrum [5]. Organic methylammonium lead iodide and formamidinium lead iodide are examples of extensively researched perovskites with the highest reported PCE of organic lead perovskite-based PV cells being 25.5% [6]. In spite of such high efficiency and simple manufacturing process, organic perovskite solar cells have the disadvantage of having toxic lead as a key element and unstable organic cations susceptible to continued exposure to light and moisture [7, 8].

To circumvent these limitations and to mitigate the harmful effects on the environment, researchers have strived to find a non-toxic, inorganic alternative for lead in solar cell absorber material [9, 10, 11, 12, 13]. Out of the viable choices for A, Cs^+^ is a promising material that has been widely studied as an inorganic alternative to organic cations like methylammonium and formamidinium [12, 14, 15]. Cesium Germanium triiodide (CsGeI_3_), in particular, has a tunable bandgap and well-balanced electron and hole diffusion lengths [11] that make it a favorable candidate for solar cell fabrication. CsGeI_3_ was practically fabricated in a stable form for the first time by Krishnamoorthy et al [16] with a PCE of 0.11%, and further work by Chen et al [17] raised the experimental PCE to 4.94%. Later Raj et al [18] numerically investigated the performance of CsGeI_3_ perovskites and reported a simulated PCE of 18.30%.

As the common device architecture of perovskite solar cells constitutes a perovskite absorber material sandwiched between an electron transport layer (ETL) and a hole transport layer (HTL), researchers are also opting for non-toxic choices for the transport layers. Zinc oxysulphide (ZnOS) is being investigated as a wide bandgap [19] alternative for the commonly used CdS buffer layers in solar cells due to the carcinogenicity of CdS. Using ZnOS buffer layer with the appropriate deposition process is expected to positively affect the stability and the efficiency of the solar cell as well as improve the *J*_*sc*_ and *PCE* [19, 20]. Korir et al [21] claimed better *PCE*, *J*_*sc*_*, V*_*oc*_ for ZnOS (11.54%, 18.50 mAcm^−2^, 0.99 V, respectively) compared to TiO_2_ (10.22%, 0.97 V, 16.50 mAcm^−2^ respectively) while comparing the performance of both materials used in the same cell structure. ZnOS has also been reported to have low toxicity, possible stability against sulphurization [22] and a variable bandgap (2.60–3.60 eV [19]), which promotes the appeal of ZnOS as a choice for window/buffer layers in PV cells. Alternatives for spiro-OMeTAD, the standard HTL candidate for high-performance PV cells, have also become desirable due to the laborious synthesis process and performance degradation of spiro-OMeTAD that creates a roadblock for cell commercialization. Both inorganic (e.g. NiO, CuI, CZTSe, Cu_2_O) and organic (e.g. Spiro-OMeTAD, P3HT, PEDOT:PSS) hole transport materials are being investigated to improve solar cell performance and stability [23].

Recent literature finds that the inorganic lead-free PSCs suffer from low PCE because of the non-radiative recombination in the bulk and interface defects (absorber-ETL and absorber-HTL interfaces). Zhang et al [24] improved the PCE to 21.18% by using the K_2_SO_4_ layer in the interface of SnO_2_ (ETL) and perovskite absorber layer as an interface defect passivation strategy. Zhuang et al [25] achieved an overall PCE of 21.31% by doping SnO_2_ ETL and reducing the interface defects using LiOH. Recently Tara et al [26] obtained a PCE of 23.1% for a CsGeI_3_ all-inorganic perovskite solar cell through numerical simulation.

This research takes the above observations into account, and aims to propose a novel all-inorganic lead-free CsGeI_3_ perovskite solar cell structure where the device parameters are optimized for the maximum possible PCE.

**Figure 1:**
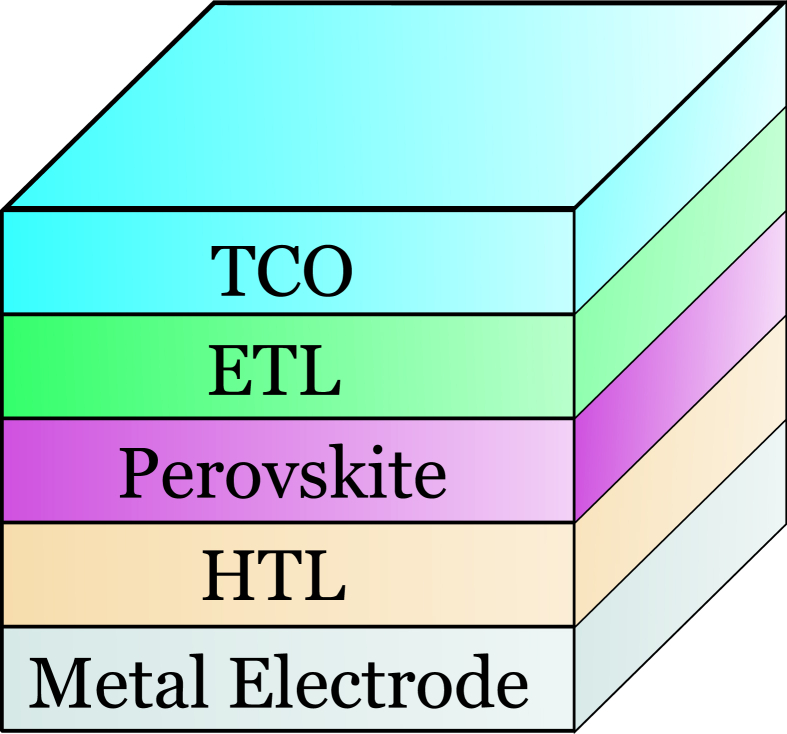
Schematic structure of proposed solar cell configuration.

As a result, a one-dimensional Solar Cell Capacitance Simulator (SCAPS-1D) [32] is used to analyze the performance of ZnOS/CsGeI_3_ solar cells numerically. First, the suggested cell structure's spectrum response is simulated for various ZnOS buffer thicknesses. Next, the effects of ETL and HTL doping concentration are investigated. In the following step of optimization, the effects of variation in absorber layer thickness on performance parameters are investigated. The impacts of bandgap tunability, interface defect density, and bulk defect density on cell performance parameters are simulated and analyzed. Finally, an optimized geometrical structure of SnO_2_/ZnOS/CsGeI_3_/CZTSe (shown in Figure 1) is proposed with the highest PCE based on simulation results.

## 2 Methodology

For efficient conversion of solar energy into useable photocurrent, the physical parameters of a solar cell are appropriately chosen to maintain proper band alignment between layers as well as charge carrier separation and blocking. The optimum thickness and doping concentration of solar cell layers and the ideal perovskite bandgap for efficient light absorption can be predicted through simulation and viable approaches can be compared prior to fabrication.

PC1D [33], SCAPS [32], wxAMPS [34], Nextnano [35], COMSOL [36], Silvaco ATLAS [37] are numerical simulators widely used in existing literature. In this work, one dimensional SCAPS-1D (v3.3.07) has been used to study the performance of the proposed solar cell. While originally developed for modelling polycrystalline CdTe and CIGS based thin film solar cells, perovskite based solar cells can also be studied in SCAPS due to their similar structure and presence of Wannier-type exciton [38]. The simulation can support up to 7 semiconductor input layers and may not be suitable for large tandem cells, but such a limitation does not negatively impact single solar cells. SCAPS also provides the option to grade almost all input parameters and specify various mechanisms and properties for recombination, defects, excitation, generation and tunneling.

The PV cell is modeled as a stack of layers defined by layer thickness, doping and various physical parameters of constituent materials. The SCAPS software calculates cell performance parameters such as *V*_*OC*_, *J*_*SC*_, *FF* and *PCE* along with material properties like band diagrams, carrier concentrations, electric fields, and currents [39] by solving three coupled differential equations, namely Poisson's equation, Eq. (1), and the continuity equations for holes, Eq. (2), and electrons, Eq. (3), under specific boundary conditions through an iterative method [18].

(1) $$\frac{\partial^{2}\psi }{\partial x^{2}}+\frac{q}{\varepsilon }\left[p\left(x\right)–n\left(x\right)+N_{D}\;–N_{A}+\rho_{p}-\rho_{n}\right]=0$$

(2) $$\frac{1}{q}\frac{\partial J_{P}}{\partial x}=-G\left(x\right)+R\left(x\right)$$

(3) $$\frac{1}{q}\frac{\partial J_{n}}{\partial x}=G\left(x\right)-R\left(x\right)$$

The approach taken by this work is to improve the performance parameters of a solar cell using the CsGeI_3_ perovskite as an absorber layer by optimizing the physical characteristics of the different layers.

## 3 Proposed structure and properties of materials

In this work, we have explored different HTL materials to find the most suitable one to get the maximum possible PCE. Table 1 shows the electrical properties of CsGeI_3_, CZTSe, ZnOS, SnO_2_, Cu_2_O, CuI, and P3HT, which are taken from different experimental and theoretical studies found in the literature.

**Table 1:** Initial dimensions and electrical properties of materials used in this simulation.

Parameters	SnO_2_ [29]	ZnOS [28]	CsGeI_3_ [18]	CZTSe [27]	Cu_2_O [30]	CuI [23]	P3HT [31]
Thickness (nm)	100	50	400	35	170	170	100
Band gap (eV)	3.6	2.83	1.6	1.4	2.17	3.1	1.85
Electron affinity (eV)	4	3.6	3.52	4.1	3.2	2.1	3.1
Permittivity	9	9	18	9	7.11	6.5	3.4
N_c_ (cm^−3^)	2.20E+18	2.20E+18	1.00E+18	2.20E+18	2.02E+17	2.80E+19	1.00E+22
N_v_ (cm^−3^)	1.80E+19	1.80E+19	1.00E+19	1.80E+19	1.10E+19	1.00E+19	1.00E+22
V_e_ (cm.s^−1^)	1.00E+07	1.00E+07	1.00E+07	1.00E+07	1.00E+07	1.00E+07	1.00E+07
V_h_ (cm.s^−1^)	1.00E+07	1.00E+07	1.00E+07	1.00E+07	1.00E+07	1.00E+07	1.00E+07
m_e_ (cm^2^.V^−1^.s^−1^)	1.00E+02	100	20	1.00E+02	200	100	1.00E-04
m_h_ (cm^2^.V ^−1^.s^−1^)	2.50E+01	25	20	1.25E+01	80	43.9	1.00E-03
N_D_ (cm^−3^)	1.00E+17	2.00E+18	-	-	-	-	-
N_A_ (cm^−3^)	-	-	2.00E+16	1.00E+19	1.00E+18	1.00E+18	3.17E+13

**Figure 2:**
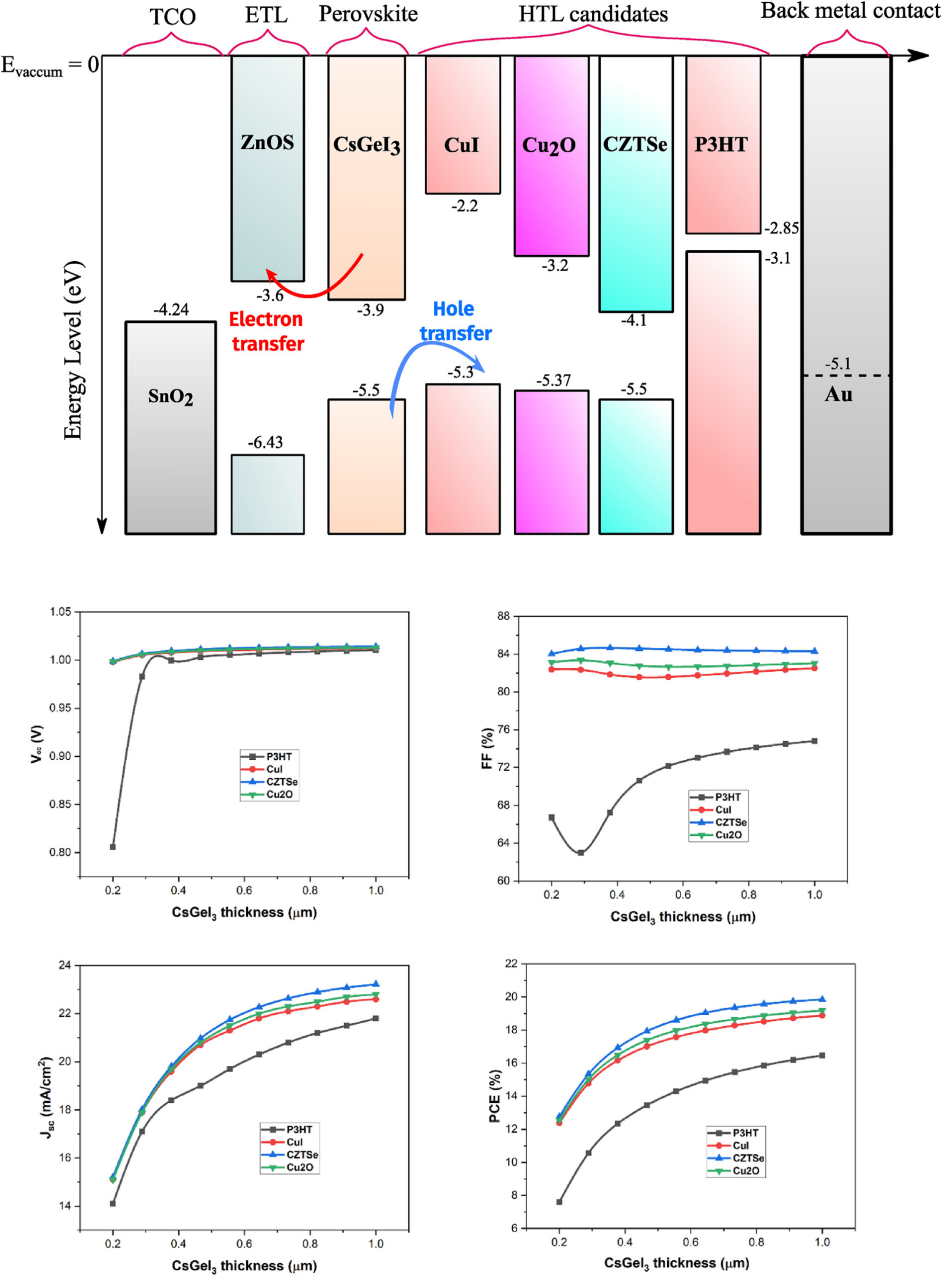
(a) Energy level diagram of the perovskite solar cell layer materials investigated in this work (including HTL candidates). (b) The effects of absorber thickness layer on the cell performance parameters.

Figure 2(a) illustrates the energy level alignments of the solar cell layers including absorber layer, ETL, different HTL and metal back contacts. The effects of absorber layer thickness on the cell performance parameters for different HTL are shown in Figure 2(b). From this figure, it is evident that CZTSe outperforms all other HTL materials. Therefore, we selected the structure configuration as SnO_2_/ZnOS(ETL)/CsGeI_3_ (absorber)/CZTSe (HTL)/Au (metal back contact) for further investigation.

For all layers, the thermal velocities of electrons and holes are assumed to equal 10^7^ cm s^−1^, respectively. CsGeI_3_ is a good light-absorbing material for solar cells, with a bandgap of 1.6 eV. The initial defect density of absorber layer is estimated to be 1×10^15^ cm^−3^ [18]. To correspond with an actual device, interface defect densities at CsGeI_3_-ZnOS and CsGeI_3_- CZTSe interfaces are also taken into account, and the simulation parameters are provided in Table 2.

**Table 2:** Initial data for interfaces between absorber and transport layers [18].

Parameters	Absorber-HTL	ETL-absorber
Defect type	Neutral	Neutral
Capture cross section area for electrons (cm^2^)	1.00×10^−15^	1.00×10^−15^
Capture cross section area for holes (cm^2^)	1.00×10^−15^	1.00×10^−15^
Energetic distribution	Single	Single
Energy level with respect to E_v_ (eV)	0.6	0.6
Characteristic energy (eV)	0.1	0.1
Total density (cm^−3^)	1.00×10^16^	1.00×10^16^

SnO_2_ and gold (back material) work functions are set to 3.6 eV and 5.1 eV, respectively. Using the initial settings from Table 1 and Table 2, cell performance characteristics such as *V*_*OC*_*, J*_*SC*_, *FF*, and *PCE* are found to be 1.010V, 20.131 mA cm^−2^, 84.649%, and 17.212%, respectively. In the following section, comprehensive investigations are carried out to investigate the impact of several key device parameters on cell performance.

## 4 Results and Discussions

### 4.1 Impact on internal quantum efficiency

The number of charge carriers generated per incident photon within the active layer of a solar cell is measured by internal quantum efficiency (*IQE*). The following equation, Eq. (4) can be used to express the relationship between *J*_*sc*_ and *IQE*.

(4) $$J_{sc}=q\int b_{s}\left(E\right)IQE\left(E\right)\mathrm{d}E$$

where, *b*_*s*_*(E)* is the incident spectral photon flux density, and q is the electronic charge.

**Figure 3:**
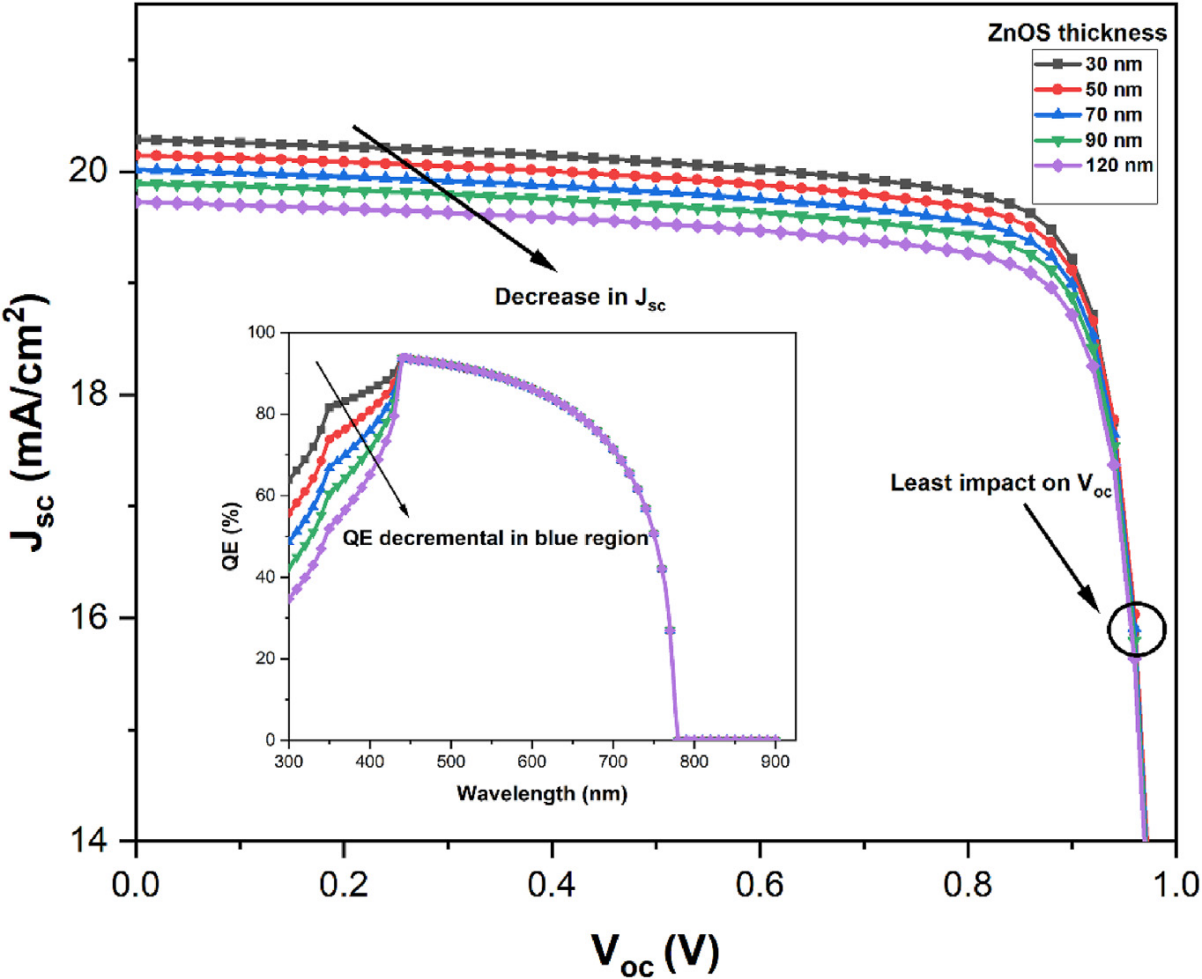
Effects of ZnOS thickness on the J-V characteristics and the spectral response of the ZnOS/CsGeI_3_ structure.

The equation clearly shows the dependency of *J*_*sc*_ on *IQE*. We have varied the thickness of the ZnOS buffer layer from 30 nm to 120 nm to explore the spectral response of the proposed cell configuration. The current density *J*_*sc*_ as a function of *V*_*oc*_ is presented in Figure 3 over this range of buffer layer thickness. It is clearly visible that increasing thickness caused a drop in *J*_*sc*_ while having a negligible impact on *V*_*oc*_. Later the *QE* versus wavelength in Figure 3 also reveals that buffer layer thickness has a considerable impact on the blue response or short wavelength region, but no deviation is observed in the long-wavelength zone.

This can be explained by the fact that most of the photons in the blue wavelength range are absorbed close to the surface; therefore, the thinner ZnOS gets closer to the surface and can collect photons more efficiently than the thicker ones [40, 41]. This explains why *J*_*sc*_ decreases due to a loss of QE% as buffer layer thickness increases, notably in the blue zone of the solar spectrum. As a result, a 30 nm thick ZnOS buffer layer film has been optimized in this study for further exploration.

### 4.2 Effect of changing doping concentration of HTL and ETL

**Figure 4:**
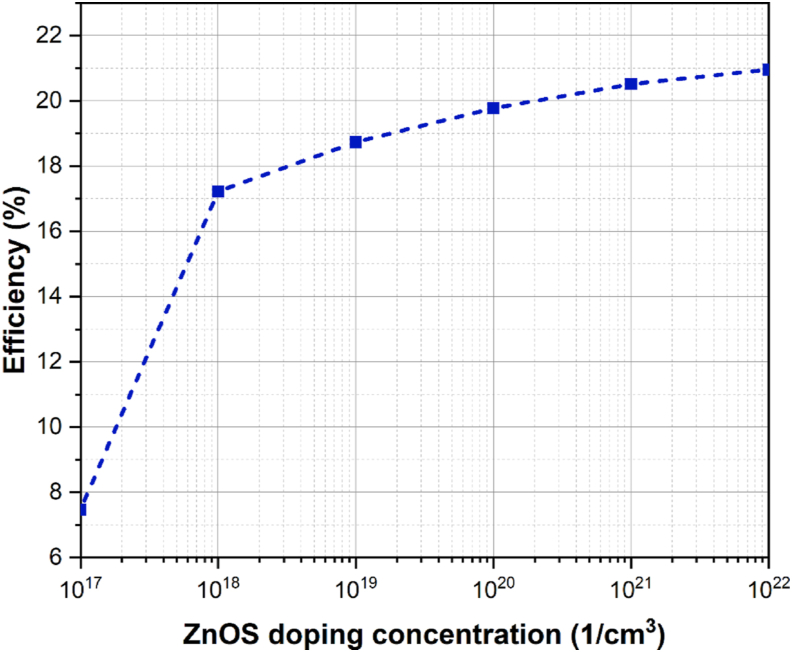
Variation in cell efficiency due to change in ETL doping concentration.

**Figure 5:**
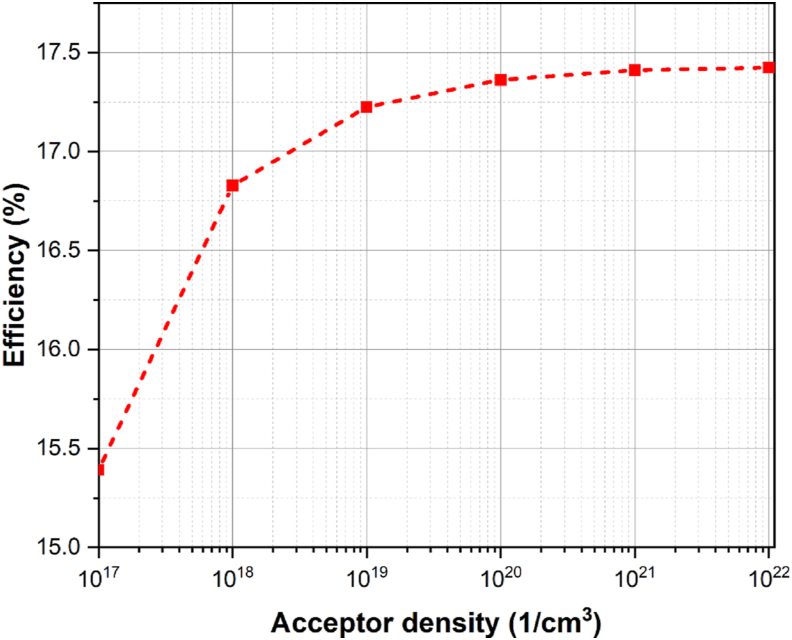
Variation in cell efficiency due to change in HTL doping concentration.

HTL and ETL doping concentrations are important factors in improving cell performance. The transport layers are in charge of transferring charge carriers created by the absorber to the external circuit. When light strikes the absorber, excitons are created [42]. The diffusion length of these excitons is longer, and the binding energy is lower [43]. After separating in the absorber layer, the charge carriers move to the appropriate electrodes and flow in the external circuit. The higher the doping, the stronger the electric field at the heterojunction interface. As charge carriers approach the HTL-absorber and ETL-absorber interfaces, strong electric fields transport holes to HTL and electrons to ETL, respectively. The electric fields at the absorber-ETL and absorber-HTL interfaces prevent electrons moving from the absorber to HTL and holes from the absorber to ETL. This phenomenon is realized from the energy level diagram shown in Figure 2(a). As a result, recombination is drastically reduced. HTL and ETL layer doping concentrations were changed from 10^15^ cm^−3^ to 10^20^ cm^−3^ and 10^17^ cm^−3^ to 10^22^ cm^−3^, respectively, and cell performance metrics were measured as shown in Figures 4 and 5. As the doping concentration rises, all parameters such as *V*_*oc*_*, J*_*sc*_*, FF*, and *PCE* rise as well. The charge carriers are swept away from the absorber layer into corresponding transport layers due to the strong built-in electric field at the interface, which causes a rise in conductivity and a fall in series resistance. As a result, more charge carriers will be able to reach the electrodes. Also, by establishing deep energy states at the junction interface, high doping concentration at the ETL-absorber lowers non-radiative recombination [43]. Due to an increase in doping in HTL, the Fermi level shifts towards the valence band, forming an Ohmic contact with the metal electrode (back metal) and allowing for efficient hole extraction in the back contact [42]. The optimal doping concentrations for HTL and ETL are 10^20^ cm^−3^ and 10^21^ cm^−3^, respectively.

### 4.3 Effect of variation of CsGeI_3_ thickness

**Figure 6:**
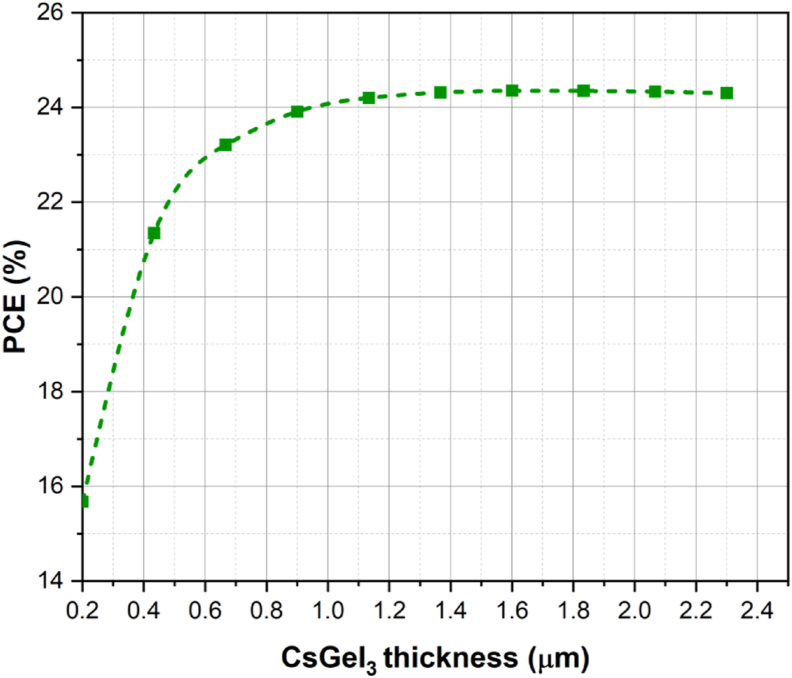
Variation of efficiency (η) with the thicknesses of CsGeI_3_.

Figure 6 shows the variation in *PCE* for different absorber layer thicknesses. Various perovskite thickness values are analyzed and found 650 nm to be the optimum value for absorber thickness as above this value, the *PCE* increment is very low.

The increase in *J*_*sc*_ and *V*_*oc*_ has resulted in an increase in efficiency. This is because by increasing the thickness of the absorber layer, more photons will be absorbed, and because the photons will be able to penetrate far enough into the absorber layer, more electron-hole pairs will be created, resulting in a higher *J*_*sc*_ and, as a result, a higher efficiency.

### 4.4 Impact of bandgap tuning

Under varying strain conditions (−4% to +4%), the band gap of CsGeI_3_ can be tuned from 0.73 eV to 2.30 eV. The Ge–I–Ge bond angle is most likely to determine the change in band gap under strain. Furthermore, the CsGeI_3_ structure is expected to remain stable in this environment [44]. The halide perovskite CsGeI_3_ achieves an appropriate band gap and optical absorption through bandgap engineering using strain, making it a promising material for solar applications.

**Figure 7:**
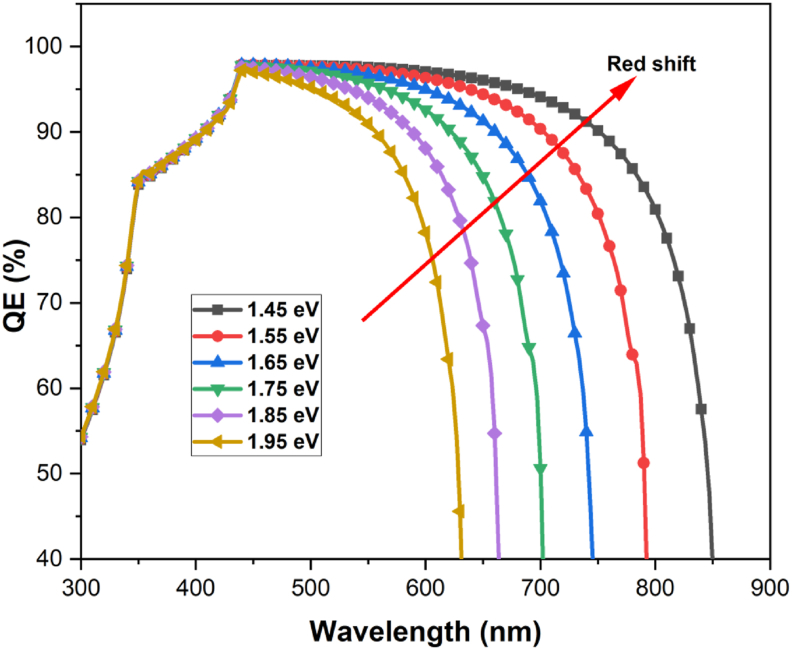
Impact of CsGeI_3_ bandgap tunability on QE of ZnOS/CsGeI_3_ cell structure.

In this work, we have established the optimal bandgap of the CsGeI_3_ layer and investigated the impact of this bandgap on the proposed structure's photovoltaic performance. The bandgap of CsGeI_3_ is varied from 1.45 eV to 1.95 eV, and the spectrum response was recorded and presented in Figure 7.

The light absorption edge of CsGeI_3_ is seen to be extended by bandgap narrowing resulting in a redshift of the CsGeI_3_ light absorption spectrum. It is clear that as the CsGeI_3_ bandgap decreases, *QE* rises steadily. Long-wavelength photons are more likely to be absorbed within the active region of the device due to the decrease in the bandgap of CsGeI_3_.

**Figure 8:**
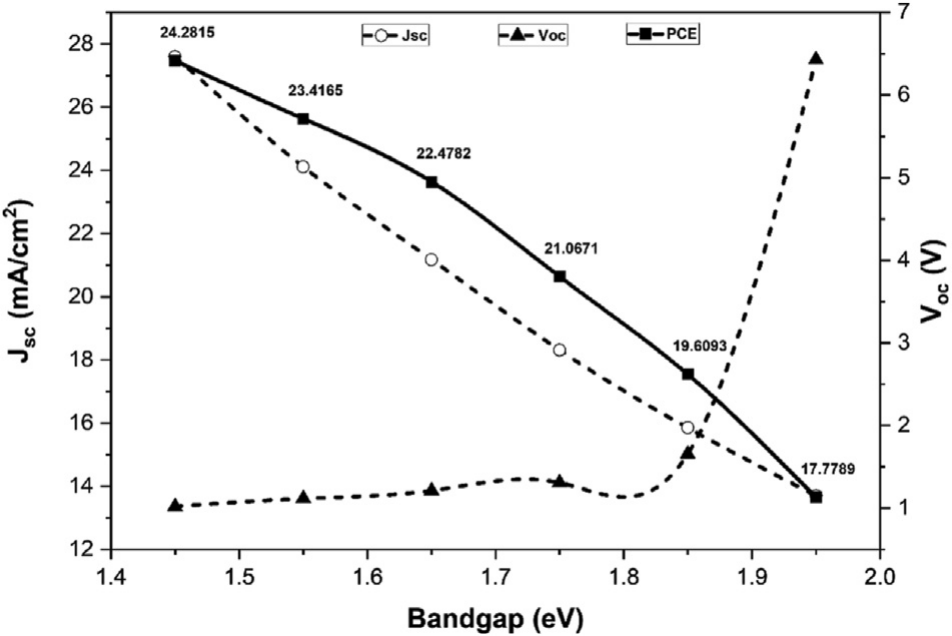
Determination of maximum PCE for the corresponding bandgap.

Figure 8 depicts the relationship between *V*_*oc*_*, J*_*sc*_, and efficiency with regard to the bandgap. E_g_ (CsGeI_3_) = 1.45 eV corresponds to *V*_*oc*_ = 1.020 V, *J*_*sc*_ = 27.596 mA/cm^2^, and maximum efficiency of 24.2815% is reached. Figure 8 depicts the fact that *V*_*oc*_ and *J*_*sc*_ follow the opposite pattern as bandgap increases. Bandgap of 1.45 eV is optimized for CsGeI_3_.

### 4.5 Impact of interface defect

The creation of cliff-type band alignment, secondary phases, and misfit dislocations can all cause recombination centers at interfaces [45]. We purposefully introduced defect states for a particular range often observed for other interfaces to explore the effect of interface defect states on solar cell performance metrics, as there is no experimental evidence of ZnOS/CsGeI_3_ structure to our knowledge. The defect state density *N*_*t*_ is modified for the range of 10^10^-10^14^ cm^−3^ in this work, which introduces an acceptor-type defect state at the ZnOS/CsGeI_3_ interface.

**Figure 9:**
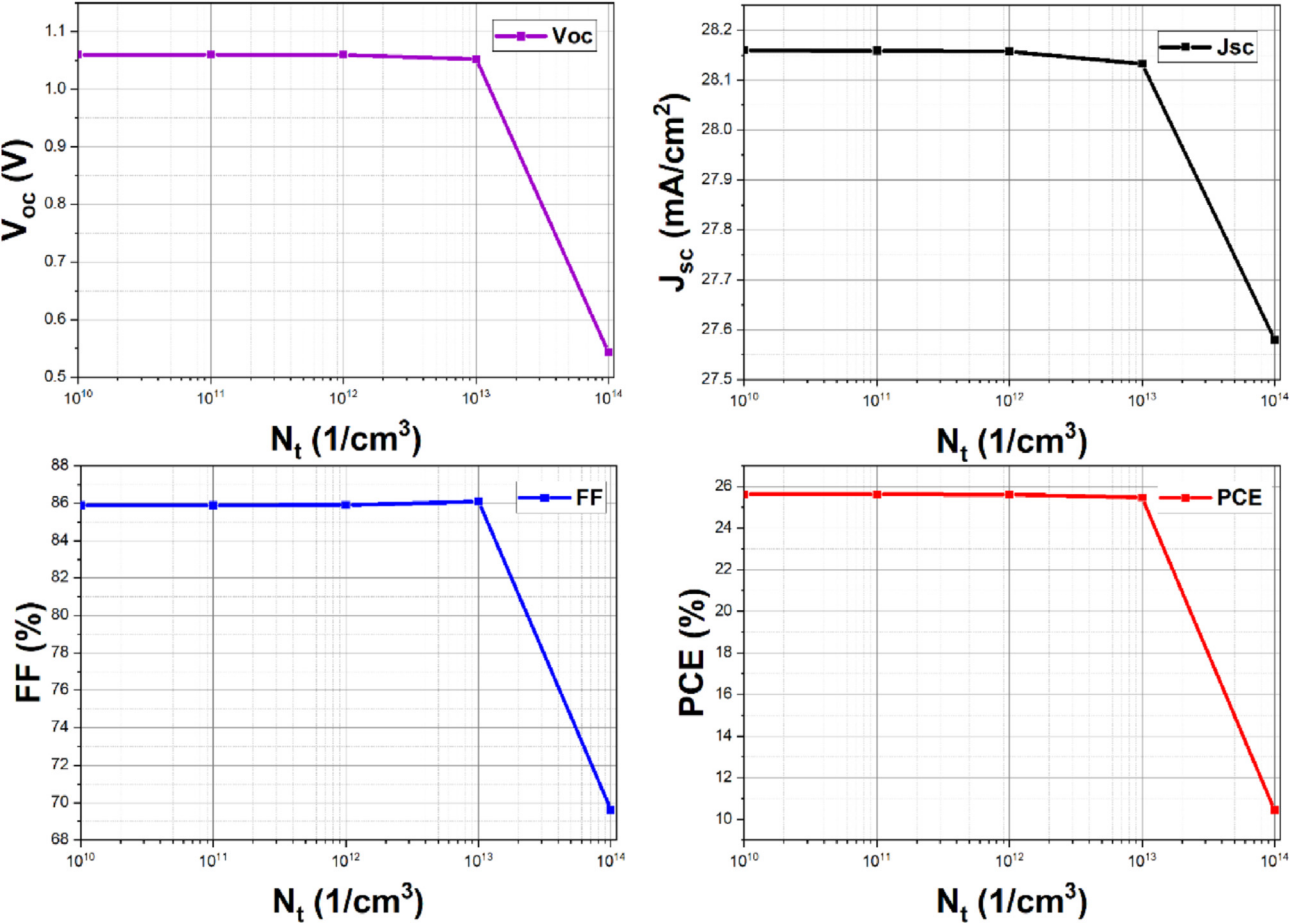
Effects of interface defect density on the solar cell parameters: V_oc,_ J_sc_, FF, and PCE.

The defect state position is theoretically moved from the bottom of the conduction band to the top of the valence band [46]. The defect states above the valence band have a stronger negative impact than the states at the conduction band's bottom [47]. Figure 9 depicts the change in solar cell performance parameters owing to an increase in defect state density.

When it comes to current density, *J*_*sc*_ remains steady at around 28.158 mA cm^−2^ for interface defect states with densities up to 10^12^ cm^−3^. Above this quantity, the defect state density increases carrier recombination at the interfaces, resulting in a rapid drop in J_sc_, as seen in Figure 9. A similar pattern can be seen in the *FF* and *V*_*oc*_ curve. A drastic fall in *V*_*oc*_ and *FF* with increasing defect densities beyond 10^12^ cm^−3^. It is due to recombination at the interface with localized energy levels. Interface flaws produce these energy levels, which reduce the *V*_*oc,*_ *J*_*sc*_*,* and the power conversion efficiency (*PCE*) of the ZnOS/CsGeI_3_ heterojunction solar cell. As a result, this 10^12^ cm^−3^ might be considered the defect state density's maximum tolerable limit.

### 4.6 Effect of bulk defect density

**Figure 10:**
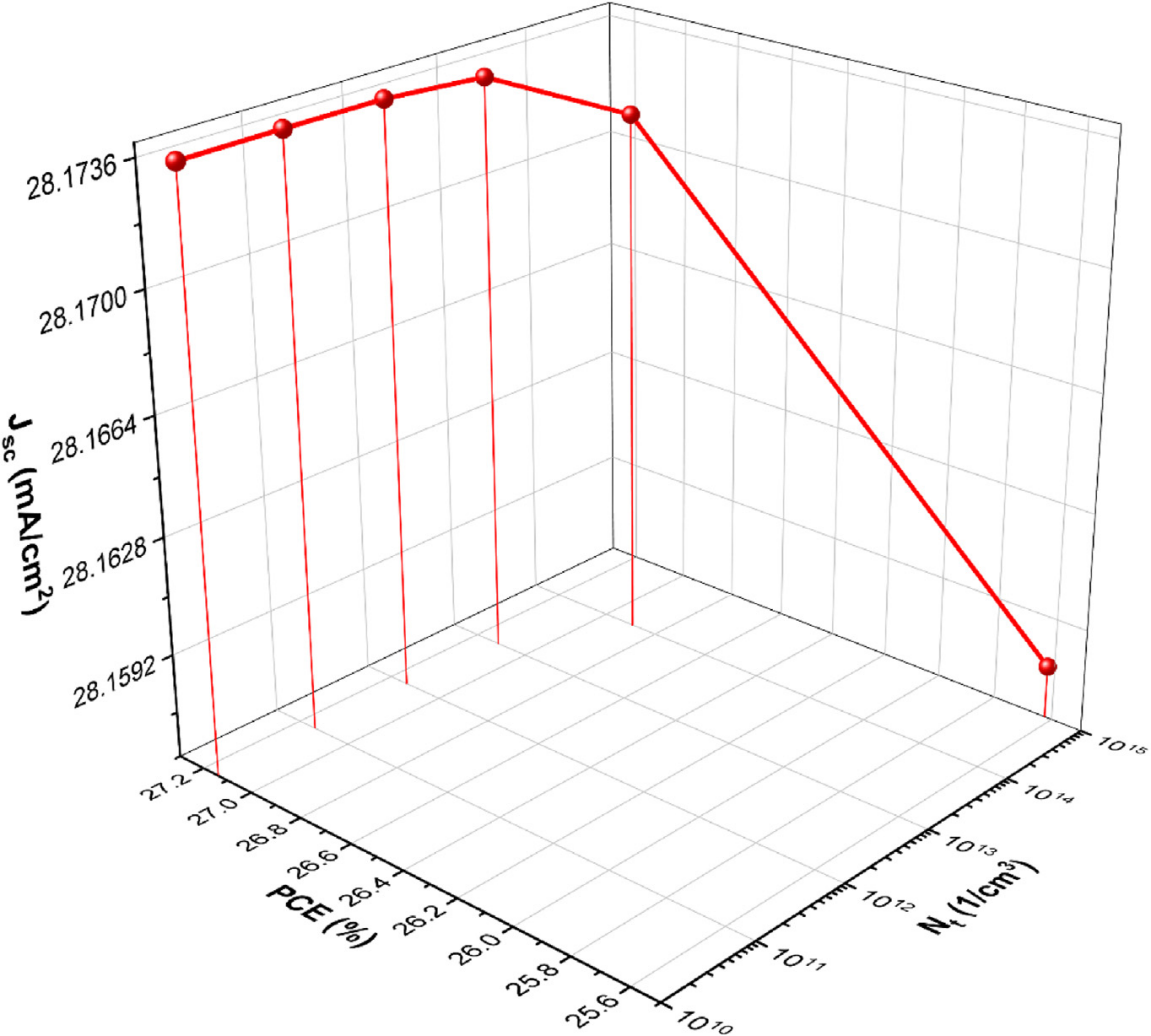
Effects of bulk defect density (*N*_*t*_) on the solar cell parameters: *J*_*sc*_ and *PCE*.

A defect in the absorber layer has a significant impact on the device's performance. To demonstrate a relationship between defect density and cell performance, the absorber defect density is varied from 10^10^ to 10^15^ cm^−3^, as shown in Figure 10. As the defect density of the absorber layer increases, the efficiency decreases significantly. At high defect density, Shockley-Read Hall non-radiative recombination shortens the minority carrier lifetime, and charge recombination occurs. As the defect density increases from 10^10^ to 10^15^cm, the PCE decreases from 26.891% to 25.624%, as seen in Figure 10. With increasing defect density in the absorber layer, IQE drops dramatically. The carrier lifetime and diffusion length increase as the defect density decreases. After 10^14^ cm^−3^, the efficiency begins to decline at a faster rate. As a result, the standard value for defect density is 10^14^ cm^−3^.

### 4.7 Effect of operating temperature

When solar cells are exposed to high temperatures, the operating temperature has an impact on their performance parameters [48, 49]. According to previous research, a rise in temperature causes a drop in open-circuit voltage (*V*_*oc*_) and a little increase in short-circuit current density (*J*_*sc*_) [50, 51, 52]. The *V*_*oc*_ and *J*_*sc*_ are affected by temperature, and their mathematical correlations are shown in equations, Eqs. (5) and (6).

(5) $$V_{OC}=nV_{T}\;\ln\left(\frac{Ip+I_{0}}{I_{0}}\right);I_{SC}≅I_{P}$$

(6) $$\frac{\mathrm{d}V_{OC}}{\mathrm{d}T}=\frac{V_{OC}-\left(V_{GO}+mnV_{T}\right)}{T}$$

**Figure 11:**
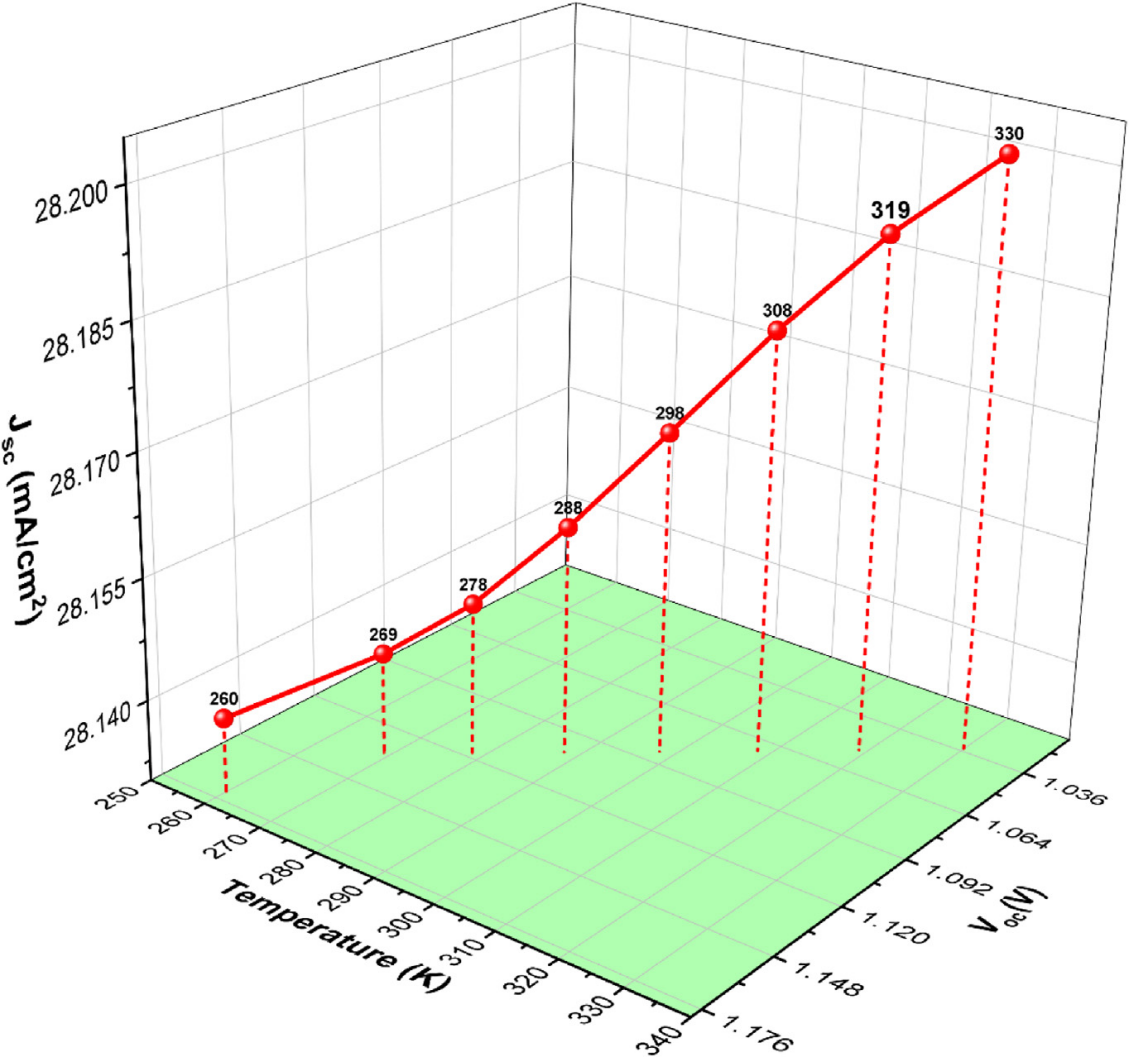
Variation of the photovoltaic parameters (V_oc_ and J_sc_) as a function of temperature.

The fluctuation of *V*_*oc*_ and *J*_*sc*_ as a function of temperature for a ZnOS/CsGeI_3_ solar cell is shown in Figure 11. With increasing temperature, the *V*_*oc*_ reduces by 2 mV/K on average, which is primarily due to the exponential increase in reverse saturation current (*J*_*00*_) [52]. It is worth noting that the *V*_*oc*_ of a Si solar cell reduces by about 2.2 mV/K as the temperature rises [44, 45]. In the instance of *J*_*sc*_, it rises by 1.43 mA per K as the effective bandgap shrinks as temperature rises [53]. The findings support that the ZnOS/CsGeI_3_ solar cell has good temperature stability.

Table 3 shows how the performance of the initial structure was improved through optimization of different parameters. Table 4 shows a quantitative comparison of this work with the experimental and simulation-based studies found in literature. The proposed optimized structure provides high efficiency at a very high fill factor. From Table 4, it is also evident that the performance parameters of this structure significantly outperform those of earlier works.

**Table 3:** Performance parameters of the cell at different optimized levels.

Parameters	*V*_*oc*_ (V)	*J*_*sc*_ (mA/cm^2^)	*FF* (%)	*PCE* (%)
Initial structure	1.0100	20.131	84.649	17.212
ETL thickness	1.008	20.269	84.602	17.288
ETL and HTL doping concentration	1.163	20.537	87.462	20.884
Perovskite thickness	1.164	22.753	87.307	23.128
Perovskite bandgap	1.020	27.596	86.237	24.282
Interface defects	1.059	28.158	85.901	25.624
Bulk defect density (absorber)	1.083	28.172	88.107	26.893

**Table 4:** Comparison of performance parameters of the optimized structure in this work with those of experimental and simulated studies.

Parameters	Chen et al	Raj et al	Saikia et al	Tara et al	This work
	[17]	[18]	[54]	[26]	
*V*_*oc*_ (V)	0.51	1.04	0.47	1.1432	1.0834
*J*_*sc*_ (mA.cm^−2^)	18.78	23.31	17.87	23.13	28.172
*FF* (%)	51	75.46	59.68	87.33	88.107
*PCE* (%)	4.94	18.3	4.99	23.1	26.893

## 5 Conclusions

In this study, the performance of SnO_2_/ZnOS/CsGeI_3_/CZTSe/Au thin-film solar cell has been numerically optimized. The optimized structure offers *V*_*oc*_ of 1.083 V, *J*_*sc*_ of 28.172 mA/cm^2^, *FF* of 88.107% and overall *PCE* of 26.893%. At *E*_*g*_ (CsGeI_3_) of 1.45 eV, a 30 nm thick ZnOS film yields the maximum power conversion efficiency of 26.893%. For various interface defect states, photovoltaic performance matrices such as *V*_*oc*_*, J*_*sc*_*, FF*, and *PCE* are simulated, and the most suitable defect density is found. The proposed cell structure provided the highest *PCE* at a defect density of 1 × 10^12^ cm^−3^. This study also explored the effect of temperature, and it is found that when the temperature rises, *J*_*sc*_ rises by 1.43 mA/K and *V*_*oc*_ falls by 2 mV/K on an average. Nevertheless, our research successfully establishes ZnOS and CZTSe as low-cost non-toxic suitable ETL and HTL materials respectively for CsGeI_3_ perovskite solar cells. In future work, the interface defect densities in perovskite solar cells may be reduced by introducing suitable buffer layers between ETL-absorber and absorber-HTL interfaces.

## Declarations

### Author contribution statement

Tasmin Kamal Tulka; Nowshin Alam; M. Mofazzal Hossain: Conceived and designed the cell structure; Performed the simulation; Analyzed and interpreted the data; Contributed analysis tools or data Wrote the paper.

Md Akhtaruzzaman; K. Sobayel: Analyzed and interpreted the data.

### Data availability statement

Data included in article/supp. material/referenced in article.

### Declaration of interest's statement

The authors declare no conflict of interest.

### Additional information

No additional information is available for this paper.
